# Exploring current trends in agricultural commodities forecasting methods through text mining: Developments in statistical and artificial intelligence methods

**DOI:** 10.1016/j.heliyon.2024.e40568

**Published:** 2024-11-20

**Authors:** Luana Gonçalves Guindani, Gilson Adamczuk Oliveirai, Matheus Henrique Dal Molin Ribeiro, Gabriel Villarrubia Gonzalez, José Donizetti de Lima

**Affiliations:** a Industrial & Systems Engineering Graduate Program (PPGEPS), Federal University of Technology - Parana (UTFPR), Via Do Conhecimento, KM 01 - Fraron, Pato Branco, PR, 85503–390, Brazil; b Expert Systems and Applications Lab, Faculty of Science, University of Salamanca, Salamanca, 37008, Spain

**Keywords:** Agricultural commodities, Agribusiness, Forecasting, Text mining, Latent dirichlet allocation, Machine learning

## Abstract

Agriculture stands as one of the major economic pillars worldwide, with food production contributing significantly to income growth. However, agricultural activities also entail risks associated with uncontrollable factors along the supply chain. To address these challenges, mathematical models have been developed for forecasting crucial variables in managing agribusiness activities. In this context, this article employs a combination of systematic bibliometric analysis and the Latent Dirichlet Allocation (LDA) method, a semi-automated approach. The main objective of this study was to automate the identification of relevant topics and construct a bibliographic portfolio (BP) covering the period 2015–2022, focusing on methodologies used in articles and other bibliometric analyses. The 30 articles included in the BP address issues related to methodologies applied in the temporal analysis of agricultural commodities. These articles were categorized based on the nature of the prediction models used, classified as (i) machine learning (ML), (ii) machine learning and artificial neural networks (ML-NN), (iii) machine learning and ensemble (ML-Ensemble), (iv) machine learning and hybrid (ML-hybrid), and (v) statistical. Regarding the results, the topic that stood out the most was termed "Forecasting Methods Applied to Agribusiness Time Series." The most utilized classes were ML-hybrid (41.95 %) and statistical (29.31 %), followed by ML-NN (14.94 %), ML (9.20 %), and ML-Ensemble (4.60 %) types. The theoretical contribution of this study lies in identifying literary gaps concerning forecasting methods applied to agribusiness, while its practical implication is to identify forecasting methodologies to support decision-making.

## Graphical abstract

**Figure:**
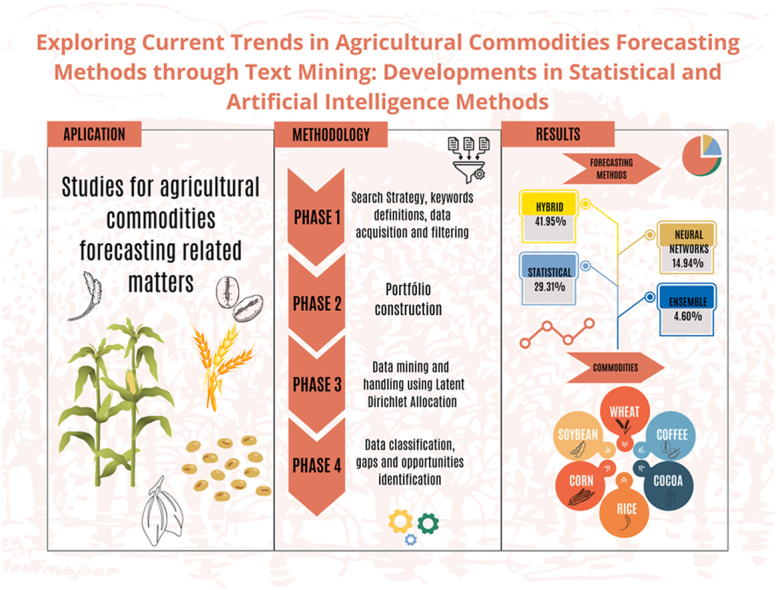

## 1 Introduction

Agriculture accounts for 4 % of global GDP and reaches a share of 25 % of GDP in developing countries. In this sense, the global agricultural sector is crucial for the generation of employment, income, and food production for approximately 9.7 billion people by 2050. Furthermore, the growth of the sector is two to four times more effective for increasing the income of the poorest communities compared to other sectors [1]. Commodities are also crucial to sustaining meat production. For example, corn is the main ingredient and source of energy in the diets commonly used in poultry farming [2].

Agribusiness is strategic and established worldwide and some variables influence the cost of commodities. There are fluctuations in the prices of agricultural commodities, which in recent years have been increasing and negatively affecting the population. If, on one hand, for the consumer, an exponential increase in prices means higher spending on food and lower well-being in general, on the other hand, it means that greater fluctuations in prices increase the uncertainty of production, resulting in increased risks to be managed [3].

In this context, the fluctuation of agricultural commodity prices is critical for farmers, governments, industries, and financial markets [4,5]. In view of this, the seasonality of agricultural product prices is an intrinsic factor to agricultural production, which can be linked to (i) excess supply in the harvest period, (ii) low supply in the off-season, and (iii) constant demand throughout the year. In addition, climatic factors also influence agricultural production, as well as pests and diseases. These factors corroborate the instability of prices by the search for the equilibrium point between supply and demand. Another relevant variable is the geographical issue, considering the same period of time, which influences the price disparity. This occurs due to the concentration of production in regional players by the transaction costs that are involved between distant regions and logistics costs [6,7].

Given the inherent risks associated with agricultural sector activities, significant strides have been made in the development of mathematical and statistical tools over the past decades, primarily focusing on price and production risk management. These tools aim to safeguard the stakeholders involved in productive activities against income fluctuations [8]. Consequently, the advent of mathematical forecasting methods has been pivotal in supporting decision-making for farmers, sectoral policies, and other stakeholders in the agricultural sector. The application of mathematical methods in price forecasting holds particular significance for commodity trading and price analysis [4,5]. Through price analysis facilitated by forecasting, farmers can mitigate risks and enhance the accuracy of decision-making in their stock negotiations. Additionally, government institutions can leverage price estimates to manage economic operations more effectively [9].

In this sense, studies on forecasting the variables of production and price in the agricultural commodities markets have been the main focus in the area [4,5]. This has occurred because it is believed that as the prices of agricultural products in the world increase, the profitability of agricultural companies and their market values tend to increase as well [10]. Thus, the analysis of the performance of the productive activity is achieved by means of forecasting the price paid to the producer, the quantity produced and the production costs. Thus, it was verified that topics such as the financial performance of the productive activity are poorly explored. To understand the studies that have already been done and the methods used, the first part of this work presents a systematic literature review using the LDA (Latent Dirichlet Allocation) method and bibliometric analysis, which aims at the literary survey of forecasting methods applied to agricultural commodities.

Recent developments in agricultural commodities price forecasting methods emphasize and justify the importance of this review.

1. Combined Models [11]: discuss the effectiveness of combining traditional and intelligent forecasting methods. This paper illustrates a growing trend toward using combined models for agricultural price forecasting. Combined approaches incorporate structured and unstructured data to improve accuracy. In the same context, the work of [12] highlights how the use of hybrid models is becoming more prevalent. Hybrid models combine traditional statistical methods with modern machine learning approaches. These models, including support vector regression (SVR) and deep learning techniques, can increase the reliability of price forecasts.
2. Machine Learning Techniques [13]: showed that extreme learning machines (ELM) outperformed other models like the traditional autoregressive integrated moving average (ARIMA) in forecasting prices for agricultural commodities. ELM, combined with genetic algorithms, has shown significant improvements in forecasting accuracy.

The research questions are: (i) What are the relevant subtopics to be studied in the context of analysis and forecasting of agricultural commodities? (ii) What are the most studied classes and methodologies for forecasting variables related to agricultural commodities? The theoretical contribution of this study was to identify gaps in the literature on forecasting methods applied to agricultural commodities.

The practical implication was to identify forecasting methodologies to support decision-making. Thus, this study presents the following contributions.

1. The manuscript provides a significant theoretical contribution by identifying gaps in the literature, specifically in forecasting methods applied to agribusiness. The focus on statistical, machine learning, and hybrid models in the context of time series forecasting for agricultural commodities is highlighted as an area with relatively sparse exploration.
2. It introduces a systematic classification of forecasting methodologies applied to agribusiness into categories like ML (Machine Learning), ML-NN (Machine Learning with Neural Networks), ML-Ensemble, and hybrid methods based on the results of [14]. This offers a structured framework for future researchers to build upon. Also, it was performed a survey of the main topics on forecasting methods of agricultural commodities and the main classes of forecasting methods applied to agribusiness.
3. A structured set of future research directions is presented for readers. These opportunities involve various strategies, including data selection, the exploration of AI techniques in agribusiness forecasting, and the types of variables to be used in forecasting systems. Additionally, the most commonly studied commodities are highlighted, revealing a significant gap that could be addressed by exploring other commodities and related variables. To achieve the aforementioned gaps, in the LDA, eight topics are labelling, those ones related to forecasting methods and strategies, variables to be forecasted, and exogenous variables incorporated in the forecasting system.

**Fig. 1:**
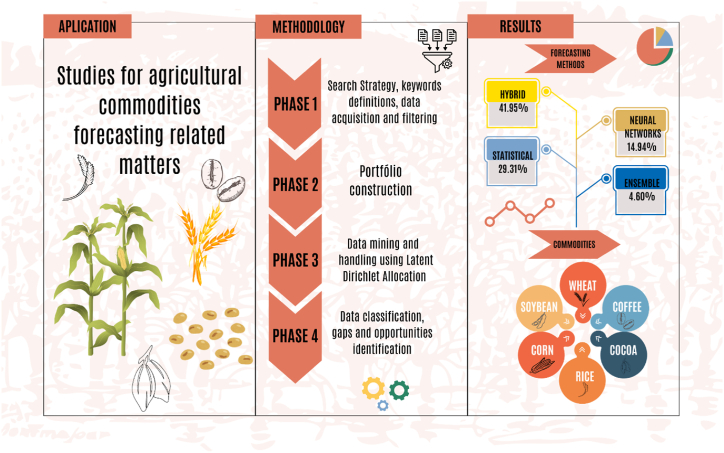
Research structure.

Source: Authors.

The remainder starts with Section 2 presenting the LDA methodology. Next, Section 3 details the methodology employed in conducting the systematic literature review. n Section 4, we present the main results of the systematic literature review, mapping out key findings and trends in the extant research literature. Following that, Section 5 discusses the research opportunities identified through the systematic literature review. Finally, i n Section 6, the concluding remarks of this study are presented, along with prospects for future research. Fig. 1 summarizes the research structure.

## 2 Latent Dirichlet Allocation (LDA)

This section presents a brief discussion on the utilization of the Latent Dirichlet Allocation (LDA) methodology. LDA is a generative probabilistic model of a corpus of text, meaning the methodology represents topics by probabilities of words, which are generated through the likelihood of words appearing in each topic. In other words, documents are represented as random mixtures over latent topics, where each topic is described through a distribution over words. Thus, LDA is an unsupervised generative probabilistic method aimed at modeling document corpora. In LDA, each document is represented as a probabilistic distribution over latent topics, and the distribution of topics in all documents commonly shares Dirichlet distributions used as prior distributions. Therefore, a corpus is denoted as D composed of M (documents), with the d (document) containing $N_{d}$ (words) (d ∈ 1, 2, …, M). Hence, LDA will model D (corpus) following the present generative process outlined below [15,16]:

i. Choose a multinomial distribution φ_t_ for topic *t* (*t* ∈ {1, 2, …, *T*}) from a Dirichlet distribution with parameter *β.*
ii. Choose a multinomial distribution *θ*_*d*_ for document *d* (*d* ∈ {1, 2, …, *M*}) from a Dirichlet distribution with parameter α.
iii. For a word *w*_*n*_ (*n* ∈ {1, 2, …, *N*_*d*_}) in document *d*,
  A. Select a topic $Z_{n}$ from *θ*_*d*_*.*
  B. Select a word $W_{n}$ from $\varphi_{zn}$.

(1) $$p\left(D|\alpha ,\beta \right)=\prod_{d=1}^{M}\int p\left(\theta_{d}|\alpha \right)\left(\prod_{n=1}^{N^{d}}\sum_{z_{dn}}p\left(z_{dn}|\theta_{d}\right)p\left(w_{dn}|z_{dn},\beta \right)\right){d\theta }_{d}\text{,}$$

Thus, the parameters α are defined by the Dirichlet topics a priori and the word distribution over topics, which, extracted from the Dirichlet distribution, give rise to *β*. Let: *T* be the number of topics, *M* be the number of documents, and *N* be the vocabulary size. The Dirichlet-multinomial pair for topic distributions at the corpus level was considered as (α, *θ*). The Dirichlet-multinomial pair for topic-word distributions was given by (β, φ). The variables *θ*_d_ are document-level variables, sampled per document. The variables $z_{dn}$ and $w_{dn}$ are word-level variables and are sampled for each word in each text document [16].

Given the above, LDA is a tool applied to uncover latent topics for a large corpus. The methodology allows the terms contained in the document set to generate a vocabulary (words), which is used for uncovering hidden topics. Documents are represented by a mixture of topics, where a topic is a probability distribution over the set of words. In this sense, each document in the corpus can be understood as a probability distribution over a set of topics. Thus, the data originate from a generative process that is defined through the joint probability distribution over what is observed and what is hidden [16].

The main positive aspect of the methodology is that LDA allows the analysis of many articles in less time, with greater transparency and replicability. LDA is a flexible tool to manage large datasets, not just for literature reviews [17]. used LDA in the context of predictions of convergence in cybersecurity technology. One of the drawbacks of topic modeling is the lack of providing a complete view of the texts, but it enables a general overview of the subtopics [18]. This occurs because LDA enables the automatic grouping of documents into homogeneous clusters, identifying the main topics of a research field. Additionally, the results are not influenced by bias and/or prior knowledge of the researcher. Thus, the results are obtained through data analysis. It is worth noting that researchers with less knowledge about data analysis can work with the methodology. Overall, the LDA methodology can be structured in four steps: (i) identification of the textual corpus, (ii) preprocessing of the text corpus, (iii) topic extraction, and (iv) topic labeling and validation [19].

Aiming to reduce bias, this article integrates systematic bibliometric analysis with an automated text mining method (LDA), resulting in a semi-automatic approach. This is due to the automation in identifying relevant topics and the ability to extract the methodologies employed in the articles, in addition to other bibliometric analyses.

## 3 Research design

The present study adapts the methodologies of [20,21]. The use of topic modeling aims to group words with similar meanings and differentiate words with multiple meanings [22]. Thus, Latent Dirichlet Allocation (LDA) topic modeling can be defined as an unsupervised probabilistic modeling method that aims to extract topics from a set of articles [[21], [22], [23]]. On the other hand, a topic can be defined as a set of words that frequently co-occur [22].

Fig. 1 presents the framework of the methodology. Step 1 involves defining the keywords. In step 2, the database sources are defined. Step 3 involves collecting the documents. A total of 388 documents (without date limitation) were returned when the keywords were entered into the ScienceDirect, Scielo, Scopus, and Web of Science search databases, accessed via the CAPES portal. Journal database sources were selected for indexing queries in academic journals and enabling information extraction [24]. Subsequently, only scientific articles from the documents generated by the platforms were filtered. Thus, in step 4, filters were applied to exclude duplicate documents. In phase 2, journal articles without duplicates were used in the LDA modeling in step 5. Later, LDA application and topic labeling were performed.

In phase 3, filters were applied to obtain articles more aligned with the research to compose a portfolio of 30 articles. The 30 selected articles underwent screening, which allowed for the extraction of the methodologies used and their categorization into five groups: (i) Statistical, (ii) Machine Learning (ML), (iii) ML-NN, (iv) ML-ensemble, and (v) ML-hybrid.

## 4 Results and discussion

**Fig. 2:**
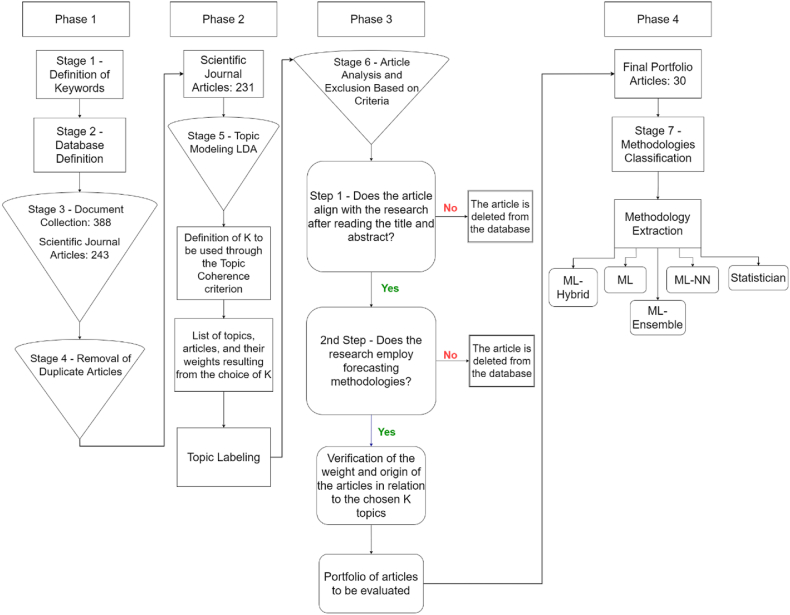
Framework of the methodology for the SLR.

Source: Authors.

In this section, the outcomes obtained in the different stages of the methodology framework used are presented, as depicted in Fig. 2.

### 4.1 Phase 1 - Document collection and filtering

**Fig. 3:**
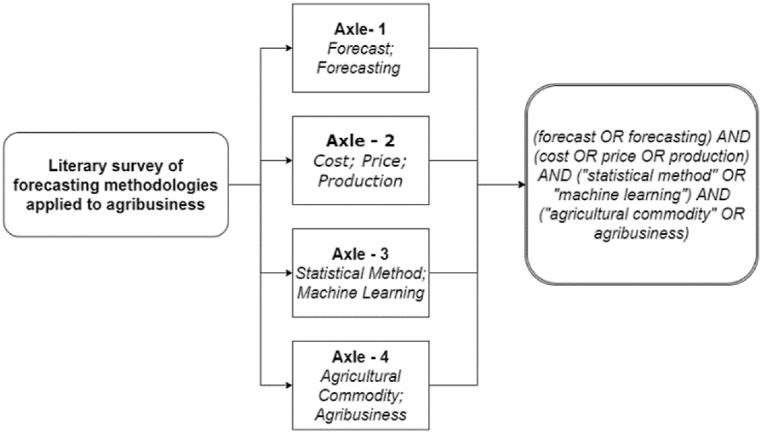
–Presentation of the keyword framework used in this study for article collection.

Source: Authors.

Fig. 3 illustrates the framework of the keywords used in this study for collecting the articles. In Phase 1, articles were gathered for analysis to achieve the objective. Axes 1 to 4, as presented in Fig. 6, were combined to standardize research across document databases. According to Fig. 2, in stage 2 of Phase 1, the database and period to be used for the literature review were defined. For this purpose, the chosen literature databases were: (i) Scopus; (ii) ScienceDirect; (iii) Web of Science; and (iv) Scielo. Thus, when entering the keywords described in Fig. 3 into the search databases, 388 documents were returned.

Consequently, non-article documents were excluded, with a total of 243 documents being filtered to remove duplicates. The first filter to exclude duplicates was done via Mendeley software, which identified 11 duplicates. Subsequently, 11 duplicate documents were removed from the article database and subjected to a second filter via visual programming, using Orange Data Mining [25], which detected one duplication. Thus, 231 articles were analyzed.

### 4.2 Phase 2 - LDA application

In Phase 2, as detailed in Fig. 3, topic modeling was implemented. While there are several ways to conduct a literature review, most of these methodologies require a significant amount of time spent reading articles, as well as pre-existing knowledge of the subject under study. Thus, the use of topic modeling presents an opportunity for researchers to expand the tools used in the literature review process. Therefore, one of the methodologies used for exploratory literature reviews is LDA, which facilitates the literature review process. Furthermore, the methodology enables a more transparent analysis, reducing the researcher's bias, which allows for review by other researchers [21].

Thus, the LDA methodology aims to analyze the words of each selected article, calculating the probability distribution among the words contained in the articles and what is in the hidden structure of the topics. Additionally, the methodology employs the 'Bag of Words' approach, which evaluates the frequency of each word without considering the semantics and meaning of the sentences. Therefore, words that are used more frequently within a topic will have a relationship with that topic [21]. Some works using LDA include: (i) the relationships between risk in financial markets and textual news information and how unstructured textual information aids in improving market volatility prediction [26]; (ii) the extraction of influential factors from agricultural futures from online news headlines [27]; (iii) the analysis of dimensions of digital transformation in the context of modern agriculture [28]; and (iv) predicting pork prices [29].

For Phase 2, topic modeling was performed using visual programming [30], employing the Orange Data Mining software based on Python. Initially, 231 articles were imported into the software. In the text preprocessing stage, an iterative list of stopping words was used, followed by the creation of word clouds for visualization and adjustment of the stopping words iteratively. LDA topic modeling was processed from K = 2 to 10 topics. It is understood that in a structured search (as depicted in Fig. 2), the limit K = 10 is reasonable because, in general, review studies address few sections or themes (in this case, topics). Fig. 3 illustrates the word cloud generated from the portfolio in preprocessing.

**Fig. 4:**
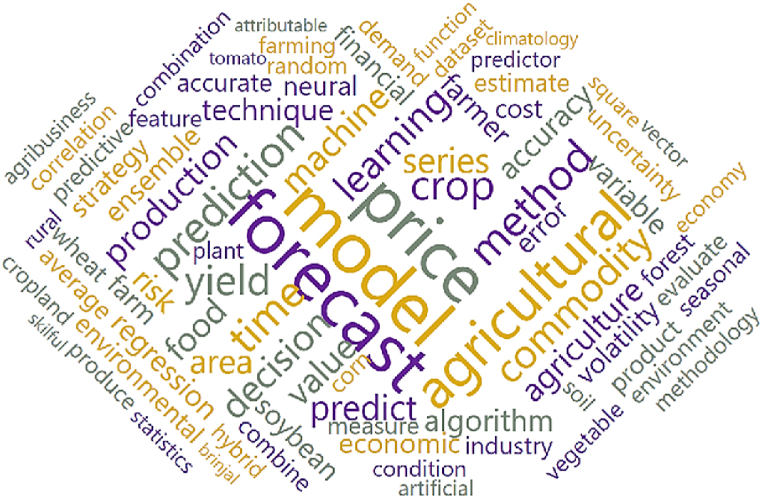
Word cloud generated from the portfolio after preprocessing.

Source: Authors.

**Fig. 5:**
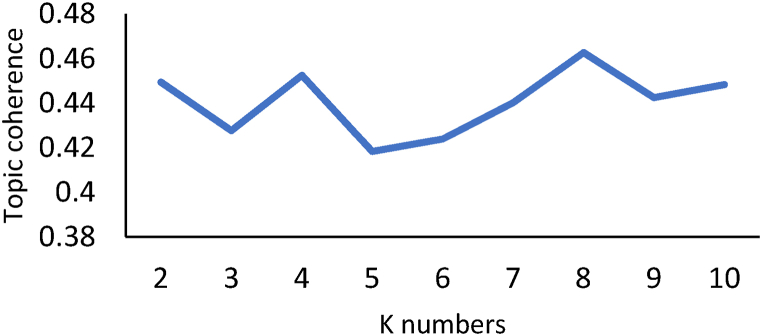
– Choice of K using topic coherence.

Source: Authors.

Through Fig. 4, it was possible to visualize the words contained in the abstracts of the 231 articles, allowing the arrival at an interactive stopping word that converged to the most appropriate value of K. With the delineation of the relevant amount of K for the research, it was possible to choose the number of topics that best aligned with the present study. The criterion used for choosing the number of topics was the topic coherence (Fig. 5) [31].

**Fig. 6:**
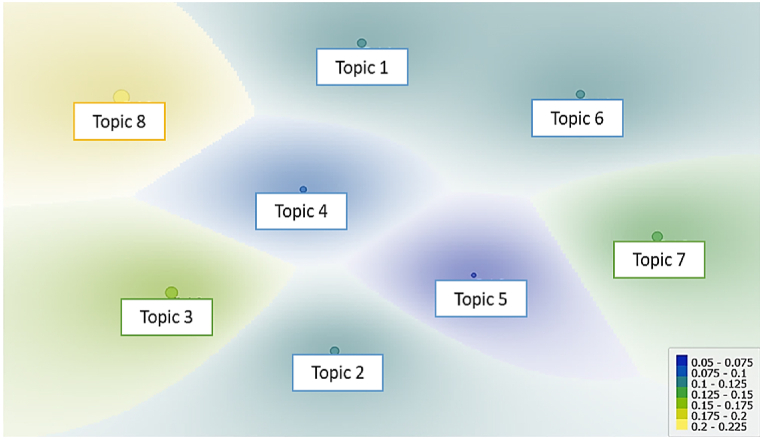
Multidimensional scaling graph (MDS) for K = 8.

Source: Authors.

From the words contained in K equals 8, it was possible to generate the topics to be analyzed. This was done by grouping words according to their frequency of appearance. In this context, Fig. 6 shows the relationship between the topics (research themes) for K = 8. The size represents how strong the topic is in the corpus of all articles. Each article has a topic weight that represents how strong each topic is in that document, i.e., LDA results are not like clustering articles.

The next step was labeling topics to identify which topics will be addressed in the remaining of the review, according to the research objectives. At this stage, a background review analysis was chosen [32], where only part of the topics generated by LDA are studied. Therefore, it was found that the theme contained in topic 8 was the most appropriate for this article, as shown in Table 1. Topic 8, labeled as Forecasting Methods Applied to Agribusiness Time Series in Tables 1 and is the most aligned with the research questions.

**Table 1:** Topics for K = 8, their respective keywords, and topic labeling.

Topic	Keywords	Description	Label
**1**	forecast, agricultural, food, method, model, decision, cost, prediction, product, accuracy	Classification methods, machine learning, and statistics for industry and agribusiness in various contexts	Classification Methods for Industry and Agribusiness
**2**	model, time, financial, series, learning, machine, correlation, ensemble, algorithm, volatility	Application of probabilistic models for climatological variables and machine learning methods for the meat industry, health, laws, and commodity volatility	Probabilistic and Predictive Methods for Agribusiness, Health, and Law
**3**	model, agricultural, commodity, forecast, learning, prediction, price, time, machine, predict	Forecasting models applied to energy commodities, water, market decision-making, the forestry market, and futures commodity markets.	Prediction Models Applied to Anergy Commodities and the Financial Market.
**4**	risk, value, model, decision, prediction, time, price, regression, forecast, strategy	Application of forecasting methods for price volatility in agricultural value chains, market risks, carbon markets, and energy.	Forecasting Methods for Price Volatility.
**5**	model, production, estimate, method, value, environment, cost, industry, environmental, product	Utilization of probabilistic models applied to artificial intelligence and use of AI techniques for industrial and agricultural problems.	Probabilistic and AI Methods Applied to Industrial and Agricultural Sectors.
**6**	crop, agriculture, agricultural, cropland, plant, farmer, farm, environmental, production, soybean	Machine learning applied to crop forecasting using historical maps of the area, remote sensing, harvest yield, projections of global grain demand and supply.	Machine Learning Methods Applied to Geoprocessing.
**7**	yield, area, model, crop, predict, wheat, production, farmer, prediction, rural	Wheat productivity forecast, commodity yield and production, weather, agriculture, nutrition, economy, and public policies.	Forecasting Methods Applied to Climate and Public Policies
**8**	price, forecast, model, series, commodity, soybean, error, method, vegetable, neural	Utilization of machine learning and time series forecasting for agribusiness.	Forecasting Methods Applied to Agribusiness Time Series.

### 4.3 Phase 3 - Final portfolio

In Stage 6 of Phase 3, articles were analyzed and excluded based on the criteria presented in steps 1 and 2. Step 1 involved checking if the article aligns with the research topic, which was done by reading the title and abstract. In Step 2, another filter was applied, requiring articles to use forecasting methods. Subsequently, the weight and topics of the articles were assessed. As a result, the final portfolio consisted of 30 articles. Table 2 shows the relationship of the journals in the final portfolio with the number of citations, acronyms, and number of publications.

**Table 2:** The relationship of the journals in the final portfolio with the acronym, and number of publications.

N°	Journal	Acronym	publications
1	Agricultural and Forest Meteorology	AFM	2
2	Applied Soft Computing	ASC	2
3	Ciência Rural	CR	1
4	Computers and Electronics in Agriculture	CEA	2
5	Energy	Energy	1
6	Expert Systems with Applications	ESA	1
7	Field Crops Research	FCR	1
8	Information Processing in Agriculture	IPA	1
9	Intelligent Systems in Accounting, Finance and Management	ISAFM	2
10	Intelligent Systems with Applications	ISA	1
1	International Journal of Forecasting	IJF	3
12	International Journal of Pattern Recognition and Artificial Intelligence	IJPRAI	1
13	International Journal of Production Economics	IJPE	1
14	International Review of Financial Analysis	IRFA	1
15	Journal of Banking & Finance	JBF	1
16	Journal of forecasting	JF	1
17	Knowledge-Based Systems	KBS	1
18	Machine Learning with Applications	MLAIJ	1
19	Neurocomputing	NC	1
20	PLoS ONE	PLoS ONE	1
21	Procedia Computer Science	PCS	1
22	Resources Policy	RP	1
23	Soft Computing	SC	1
24	The North American Journal of Economics and Finance	NAJEF	1

According to Table 2, the journal that stood out the most in the portfolio in terms of publication quantity was the International Journal of Forecasting, representing approximately 10 %, followed by the journals Intelligent Systems in Accounting, Finance and Management, Computers and Electronics in Agriculture, Applied Soft Computing, and Agricultural and Forest Meteorology, each with a participation of around 6.67 %. The remaining journals represented approximately 3.33 % each. Fig. 6 presents the dynamics among the top 10 most cited authors, the journals, and the databases in which these articles were published.

**Fig. 7:**
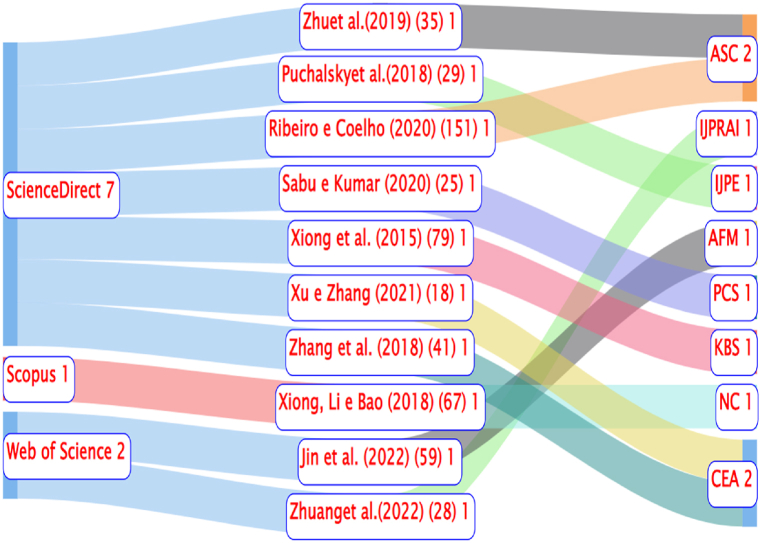
Dynamics of the top 10 most cited authors, the journals, and the databases in which these articles were published.

Source: Authors.

According to Fig. 7, the most cited authors and their respective citations were: (i) Ribeiro and Coelho (2020) (151) [33]; (ii) Xiong et al. (2015) (79) [34]; (iii) Xiong, Li, and Bao (2018) (67) [35]; (iv) Jin et al. (2022) (59) [36]; (v) Zhang et al. (2018a) (41) [37]; (vi) Zhu et al. (2019b) (35) [38]; (vii) Puchalsky et al. (2018) (29) [39]; (viii) Zhuang et al. (2022) (28) [40]; (ix) Sabu and Kumar (2020) (25) [41]; and (x) Xu and Zhang (2021a) (18) [42]. The target journals were authors Ribeiro and Coelho (2020) [33] and Zhu et al. (2019b) [38] published in Applied Soft Computing. Meanwhile, authors Zhang et al. (2018a) [37] and Xu and Zhang (2021a) [42] published in Computers and Electronics in Agriculture. Overall, for the portfolio of 30 articles, the participation of databases was as follows: (i) ScienceDirect (70 %), (ii) Scopus (13.33 %), (iii) Web of Science (13.33 %), and (iv) SciELO (3.33 %).

The top 10 most cited articles were examined regarding the forecasting methodologies and applications. The standout article was by authors Ribeiro and Coelho [33], which aimed at evaluating the predictive capacity of ensemble models comparing them with each other and approaches that assess models uniquely, which are reference models. Additionally, a one-year ahead forecast horizon was adopted, using monthly time series data regarding prices paid to producers in the state of Paraná, Brazil, for a 60 kg sack of soybeans and wheat. In this context, the methodologies used were the ensemble regression sets gradient boosting machines (GBM), eXtreme Gradient Boosting (XGBoost), random forest (RF), and stacking ensemble (STACK), with multilayer perceptron (MLP), support vector regression (SVR), and k-nearest neighbors (KNN) as reference models. The performance measures used were mean absolute percentage error (MAPE), root mean square error (RMSE), mean absolute error (MAE), and mean squared error (MSE). Moreover, Friedman and Wilcoxon tests were employed to evaluate the absolute percentage errors (APE) of the models. The results indicated that ensemble models, especially the XGBoost model, exhibited superior performance to single models. Furthermore, boosting-based ensemble approaches showed robust performance for both commodities, with MAPE < 1 % and high accuracy.

Consequently, the article by Xiong et al. (2015) [34] aimed to forecast cotton and corn prices. Data were obtained from the Chinese futures market with daily quotations. Forecast horizons were one year ahead (h = 1) and multi-horizons (h = 3,5). Additionally, "linear and nonlinear" modeling was followed, proposing a new methodological approach. The methodologies used were vector error correction model (VECM) and multi-output support vector regression (MSVR), which formed the VECM-MSVR model, compared with ARIMA-MSVR, ARIMA-NN, VECM, SSVR, and MSVR models. The MAPE was used as the metric to measure model accuracy. The results indicated that the proposed method VECM-MSVR outperformed the other five, suggesting it as an alternative for futures market prediction.

Authors Xiong, Li, and Bao (2018) [35] aimed to propose a new hybrid method, combining seasonal trend decomposition based on loess (STL) and extreme learning machines (ELMs). They analyzed the seasonality behavior of vegetable prices, namely cabbage, pepper, cucumber, green beans, and tomatoes from the Chinese agricultural market, in the short, medium, and long terms (h = 1, 3, and 6). The database comprised monthly prices of 5 vegetable price series from January 2002 to April 2014. The ELM method was compared with SARIMA, time-delay neural network (TDNN), SVR, and SARIMA-Kalman filter. Symmetric mean absolute percentage error (SMAPE) was used to measure accuracy. Results for the STL-ELM model was 3.217, 3.231, 3.197, 3.185, and 3.205 for vegetable forecasts. Thus, the proposed STL-ELM model showed better performance compared to others, considering short, medium, and long-term horizons. According to the authors, this method is more suitable for forecasting vegetable prices with high seasonality.

Jin et al. (2022) [36] utilized seasonal climate forecasts (SCF) to predict winter wheat yield under water constraints in Australia. The methods employed were Seasonal Climate Prediction (SCF) and a classical downscaling technique (QM) to drive the APSIM biophysical model. They also utilized the newly developed extended copula post-processing technique (ECPP) and the Schaake shuffle to downscale four climate variables to seasons, to examine individual climate variables and their cross-dependence. Accuracy measurements for the methodologies included bias and spread skill in ensemble propagation, correlation and root mean square error (RMSE), relative change and spread of ensemble forecast, and ranked continuous probability score (CRPS). ECPP yield predictions showed significant improvement over quantile mapping downscaling and raw SCF from the recent Australian seasonal prediction model ACCESS-S1, demonstrating enhanced forecasting capability. Moreover, even at the beginning of a season with a forecast lead time of 4 months or more, ECPP-driven yield predictions outperformed. Additionally, early-season yield predictions driven by SCFs provide an alternative to machine learning-based forecasts.

Zhang et al. (2018a) [37] analyzed Chinese soybean prices using a monthly price dataset from January 2010 to December 2015, aiming to propose a model of quantile regression radial basis function neural network (QR-RBF). To optimize the QR-RBF neural network model parameters, they utilized the hybrid GDGA method based on a combination of genetic algorithm and gradient descent. Results showed that the author’s proposed model achieved satisfactory results, with the highest absolute value of ER at 1.79 % and MAPE at 1.11 %.

Zhu et al. (2019b) [38] proposed a hybrid model called VMD–BiGRU to forecast daily prices and volatility of natural rubber traded on the Shanghai futures market. The methods used included simple GRU and BiGRU models for daily closing price predictability and 7-day volatility, as well as the hybrid model (VMD–BiGRU). GRU, BiGRU, and VMD–BiGRU models were compared for a 1-day forecast horizon. Subsequently, high-frequency, medium-frequency and low-frequency subsequences were inputted into the VMD-BiGRU hybrid model to generate single-feature hybrid models (VMD-H-BiGRU, VMD-M-BiGRU, and VMD-L-BiGRU) for closing price and 7-day volatility prediction. Accuracy measurement models included statistical methodologies like R-squared (R2), RMSE, MAPE, and DA (Directional Accuracy). Results indicated that the variational mode decomposition method was effective for time series analysis, bidirectional neural network structure improved model performance, and an intrinsic correspondence between price and volatility was found.

Puchalsky et al. (2018) [39] aimed to evaluate the performance of Wavelet Neural Networks (WNNs) combined with five optimization techniques. Two studies were conducted: the first analyzed soybean prices and the second addressed the demand issue of distinct product groups from a food company. Optimization techniques included: Differential Evolution, Artificial Bee Colony, Glowworm Swarm Optimization, Gravitational Search Algorithm, and Imperialist Competitive Algorithm. Analysis horizons were set at 30 days for soybean prices and 12 months for product demand. The optimization techniques' performance in training WNN was compared with backpropagation and Extreme Learning Machine (ELM) algorithms. Results showed that for long-term forecasting, different methods achieved the best accuracy according to each case. Additionally, the forecast horizon influenced the optimization methods' performance, as well as the WNN learning process optimization.

Zhuang et al. (2022) [40] utilized long short-term memory (LSTM) and artificial neural network (NN) methodologies. Prediction methods were applied to rice, wheat, corn, soybean, beef, and pork commodities, aiming to forecast price, production, consumption, import, export, and balance variation values. Forecast accuracy measurement methods included MSE and MAE. The study concluded that the LSTM deep learning method contributed to improving the accuracy of the multivariate coupling model by at least 15 %, enhancing the forecast of agricultural commodity supply and demand.

Sabu and Kumar (2020) [41] employed the Seasonal Holt-Winter Method, SARIMA model, and machine learning model - LSTM, for areca nut price prediction using a monthly price dataset covering the period from 2007 to 2017. Results were compared among different methods, and model performance was assessed using RMSE. The conclusion was that the LSTM neural network model was the best for areca nut price forecasting.

In Xu and Zhang's study (2021a) [42], the naive model, linear autoregressive model (LAR), NAR, and NARX prediction methods were utilized. These methods were applied to a daily corn price dataset from 500 markets across 16 North American states. Models were compared, and performance was evaluated using MSE and Mahalanobis distance matching (MDM). It was concluded that simple neural networks with 20 hidden neurons and 2 lags showed the best performance for a 1-day forecast horizon.

### 4.4 Phase 4 - Classification of mapped methodologies

**Fig. 8:**
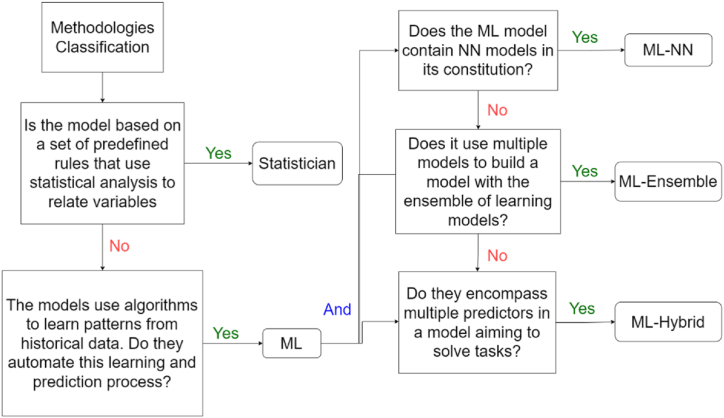
The relationship between topics and the research theme for k = 8.

Source: Authors.

The focus is on methodologies for agricultural commodity forecasting. The portfolio underwent two additional refinements in phase 3 (Fig. 2), excluding articles based on title and abstract and aligning with the study's subject matter and methodologies. These articles primarily focused on extracting methodologies. In phase 4, methodologies were categorized based on the criteria in Fig. 8:

Fig. 8 illustrates the classification of methodologies extracted from the articles in the final portfolio. The definitions were: (i) Statistical Methodologies; (ii) Machine Learning (ML) methodologies [43,44]; (iii) Machine Learning – Artificial neural network (NN) methodologies [45]; (iv) Competitive Methods or ML-Ensemble; and (v) Collaborative Methods or ML-Hybrid [14].

In summary, the following forecasting methods are identified: (i) statistical methods are based on a set of predefined rules that use statistical analysis to relate variables. Here it is possible to highlight the approaches: Box-Jenkins, Holt Methods, dynamic regression, exponential smoothing, Naïve approach, and their variations; (ii) Machine Learning (ML) methods: These methods use algorithms to learn patterns from historical data. They automate this learning and prediction process [46]; and (iii) ML-NN: are those models that contain Neural Network models in their constitution [45].

In general, ensemble models are machine learning algorithms that build a set of classifiers and subsequently classify new data points through a weighted vote of the predictions [47]. These models are a paradigm of ML and are achieved through training different algorithms to solve complex issues of the model network. Thus, models are combined to maximize prediction accuracy [48]. The first method used was Bayesian averaging, and subsequently, new approaches were used such as error-correcting output coding, bagging, and boosting. Bagging, boosting, and stacking techniques are considered classical, serving as a basis for the development of others [49]. Other ensemble methodologies used include voting and AdaBoost [50].

**Fig. 9:**
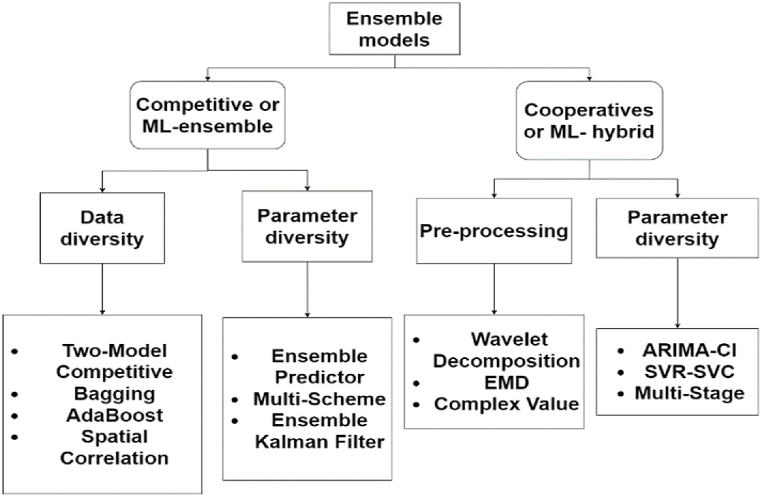
Framework of the subdivisions of ensemble method classes.

Source: Adapted from Ren, Suganthan, and Srikanth [16].

Ensemble models can be divided into two classes: (i) competitive methods and (ii) collaborative methods [14]. Thus, collaborative methods can be classified as ML-hybrids and competitive models as ML-Ensemble [46], as shown in Fig. 9, which illustrates the subclassifications of ensemble models.

In general, to generate forecasts using ensemble methods, four strategies are necessary: (i) data diversity, (ii) parameter diversity, (iii) kernel diversity, and (iv) preprocessing diversity [51]. From this perspective, competitive methods with two subcategories are (i) data diversity, exemplified by the competitive towing model, Bagging, AdaBoost, Spatial correlation, and (ii) parameter diversity, exemplified by Ensemble predictor, Multi-Scheme, Ensemble Kalman Filter. Meanwhile, cooperative or hybrid models have subclasses: (i) preprocessing: Wavelet Decomposition, Ensemble empirical mode decomposition (EMD), Complex Value, and (ii) post-processing: ARIMA-Computational intelligence (CI), SVR-Support vector classification (SVC), Multi-Stage [14].

Competitive methods or ML-Ensembles are models that integrate prediction results from various models, meaning multiple models are used to build a model with a learning model set [[46], [47], [48], [49], [50], [51], [52]]. Diversity is a fundamental characteristic of competitive models, achieved through data, parameter, and kernel diversity. Thus, competitive models make predictions by training different predictors individually, using the same or different datasets and different parameters. Subsequently, the prediction is obtained by averaging or otherwise combining the individual predictions of the base predictors [14]. Some examples of competitive models are: (i) RF [53], (ii) GBM [54], (iii) XGBoost [33], and stacking and its variations [46].

Collaborative methods or ML-Hybrid are cooperative models that encompass multiple predictors in one model aiming to enhance prediction accuracy and minimize model errors. In this regard, cooperative ensembles or hybrid methods generate predictions by dividing prediction tasks into several subtasks, and the most suitable predictor is selected based on the characteristics of these subtasks. Additionally, the final prediction is obtained by summing the outputs of the base predictors [14], summing the individual results of time series preprocessing methods [55], using metaheuristics for model hyperparameter tuning [56], feature selection [57], statistical models [58], and AI models [59].

Hypertuning defines the best configuration of the model's structure, thus improving its predictive capacity [60]. The major advantage of this approach is that the adjustment is carried out through multi-criteria optimization, thereby the combination of hyperparameter adjustments results in an overall improvement in the structure [61]. One of the ways to improve prediction performance is to reduce noise [62], which can be done using techniques based on the wavelet transform [63], empirical wavelet transform [64], variational mode decomposition [65], seasonal decomposition approaches [66], Hodrick–Prescott filter [67], or Christiano-Fitzgerald random walk filter [68]. The inclusion of decomposition techniques to reduce noise produces hybrid models with greater predictive capacity [69].

**Fig. 10:**
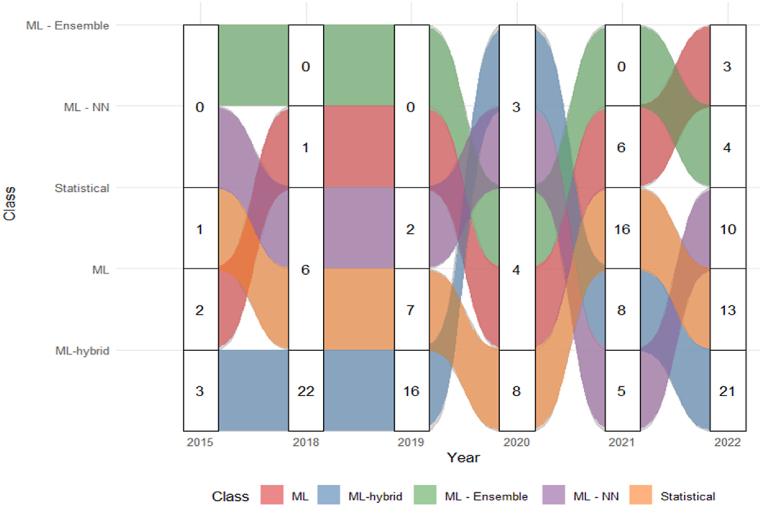
The relationship between class usage over time.

Source: Authors.

One of the differences between these two ensemble classes is the computational cost required, where cooperative methods demand less computational power compared to competitive ensembles. This is because cooperative ensembles require less modeling of a larger dataset from different stations, as well as modeling a dataset using various parameter sets. Thus, the forecasting horizon of cooperative ensembles is generally shorter compared to competitive ensembles, making them more suitable for short-term predictions. On the other hand, competitive models are suitable for medium to long-term horizons, as they require a greater number of datasets or a larger number of parameters for prediction [14]. In this regard, the methodologies extracted from the 30 articles of the final portfolio were classified as (i) ML, (ii) ML - NN, (iii) ML - Ensemble, (iv) ML-hybrid, and (v) statistical. Fig. 10 highlights the relationship of class usage over time.

According to Fig. 10, the year 2022 represented approximately 29.31 % of the methodologies, while the years 2021 and 2018 depicted about 20.11 % of the methodologies used. Subsequently, the year 2019 represented 14.37 %. The year 2020 ranked fifth with 12,64 % of the methodologies used. Lastly, the year 2015 only obtained about 3.45 % of the methodologies used. Additionally, during the analyzed period, hybrid ML methodologies were the most used, representing approximately 41.95 % of the methodologies. The second most used class was statistical (29.31 %). Consequently, ML-NN methodologies represented 14.94 %, followed by ML methodologies with the participation of 9.20 %, and finally, ML-Ensemble methodologies with only 4.60 % utilization in the final portfolio articles.

In 2015, hybrid ML methodologies represented about 50 % of the methodologies used, followed by ML (33.33 %) and statistics with 16.67 %. In this context, in 2018, hybrid ML models were the most used, representing about 62.86 % of the methodologies. Subsequently, statistical methods and ML-NN had a participation of 17.14 %, and ML models obtained only 2.86 % of the usage. In 2019, the use of hybrid ML models (64 %) was the most predominant compared to other methodologies, with statistics having the participation of 28 % and ML-NN with 8 %.

**Fig. 11:**
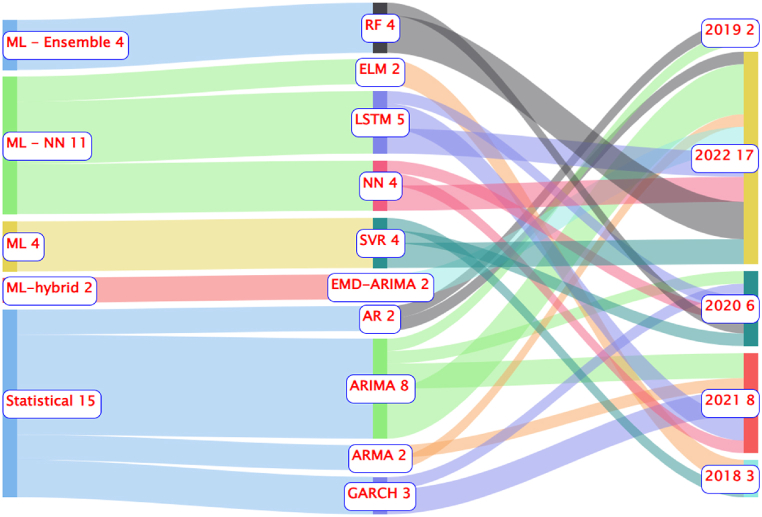
The top 10 methodologies and classes used.

Source: Authors.

In 2020, statistical methods (36.36 %) were the most used. Subsequently, ML-Ensemble and ML had a participation of 18.18 %. The hybrid ML and ML-NN methods represented 13.64 % of the methodologies used. In 2021, statistical methodologies (45.71 %) had the highest participation compared to others, with hybrid ML methodologies at 22.86 %, followed by ML with 17.14 %, and ML-NN with 14.29 %. In 2022, hybrid ML methods returned to prominence with 41.18 % usage, followed by statistical (25.49 %) and ML-NN with 19.64 %. Meanwhile, ML (5.88 %) and ML-Ensemble (7.84 %) had the lowest participation in the year. Fig. 11 presents the top 10 methodologies and classes used.

**Fig. 12:**
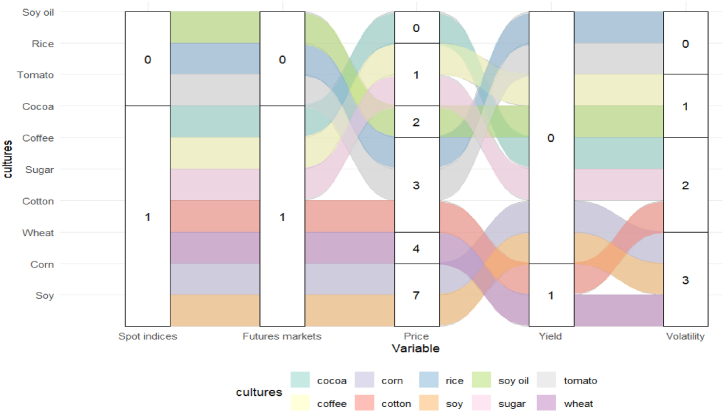
Dynamics among the top 10 cultures and the 5 most studied variables.

Source: Authors.

Just like Fig. 11, in the ranking of the 10 most used methodologies, the statistical class stood out the most with 41.67 % of participation among the classes, so the participation of methodologies belonging to the class in relation to the total methodologies was: (i) ARIMA (22.22 %); (ii) GARCH (8.33 %); (iii) ARMA (5.56 %); and (iv) AR (5.56 %). The ML-NN class (30,56 %) was the second most used, with the methodologies that stood out from this classification being: (i) LSTM (13.89 %); (ii) NN (11.11 %); and (iii) ELM (5.56 %). From the ML-Ensemble class, which represented about 11.11 %, the most used method was RF (11.11 %). In the ML class, which had a participation of 11.11 %, the method that stood out the most was SVR (11.11 %). The hybrid ML class represented about 5.56 % among the classes, and the most used method of this class was EMD-ARIMA with 5.56 %. Fig. 12 illustrates the dynamics among the top 10 methodologies and their studied variables.

Thus, the most exploited commodities among the top 10 are soybean and corn crops and were studied in conjunction with the following variables: (i) spot indices (8.33 %); (ii) futures markets (8.33 %); (iii) price (58.33 %); and (iv) volatility (25 %). Additionally, studies on wheat commodity focused on the variables: (i) spot indices (10 %); (ii) futures markets (10 %); (iii) price (40 %); (iv) yield (10 %); and (v) volatility (30 %). Studies on cotton crops focused on the following variables: (i) spot indices (12.50 %); (ii) futures markets (12.50 %); (iii) price (37.50 %); (iv) yield (12.50 %); and (v) volatility (25 %).

For sugar commodity, the studied variables were: (i) spot indices (20 %); (ii) futures markets (20 %); (iii) price (20 %); and (iv) volatility (40 %). The involvement of variables in cocoa crops was: (i) spot indices (25 %), (ii) futures markets (25 %), and (iii) volatility (50 %). As for coffee crops, the studied variables were (i) spot indices (25 %), (ii) futures markets (25 %), (iii) price (25 %), and (iv) volatility (25 %). Unlike other commodities, rice and tomato crops focused solely on the price variable in the studies. As for soybean oil commodity, the most important variables were prices (6667 %) and volatility (33.33 %).

### 4.5 Discussion and research opportunities

We identified only two prior studies that also explored forecasting methods for agricultural products, utilizing a review as their primary research method (Table 3).

**Table 3:** – Literature reviews on forecasting methods for agricultural commodities.

Study	Summary	Main Findings
Agricultural product price forecasting methods: research advances and trend [70].	Reviewing procedures described with a research design section. Text mining and LDA used to explore the topics on the	The main findings suggest that future research should focus on the use of hybrid models for predicting agricultural product prices. Also, it is necessary to expand the application of models that account for price-influencing factors. Moreover, model performance should be evaluated with decision-support metrics rather than relying solely on error-based measures. Finally, seasonal adjustment models may enhance the accuracy of forecasts in complex seasonal trends, and hybrid optimization algorithms can be employed to further improve prediction performance.
Agricultural Product Price Forecasting Methods: A Review	Reviewing procedures not clearly described as there is not a research design section. Text mining and LDA not used or not mentioned.	The main findings indicate that the use of combined models for forecasting agricultural product prices represents a key future development. Understanding the principles of model combination is essential for achieving accurate predictions. Another promising trend is the integration of structured and unstructured variable data into price forecasting models. Furthermore, ensuring both the accuracy of predicted values and the precision of forecasted trends will be essential in future research on agricultural product price prediction.

In comparison to the previous works in Table 3, our article takes a more retrospective approach, providing a comprehensive perspective of current forecasting methodologies in agribusiness, whereas the other texts are suggesting future directions for model development. While both approaches are valid, our paper contributes to this research field by systematically analyzing the literature and identifying gaps, providing a solid foundation for future studies.

Another key difference is that our research design applies Latent Dirichlet Allocation (LDA) and text mining techniques to automate the review process, efficiently extracting and categorizing thematic patterns from a large body of research. This semi-automated approach enables the identification of key topics and trends in forecasting, making it a valuable tool for organizing and interpreting complex datasets.

**Fig. 13:**
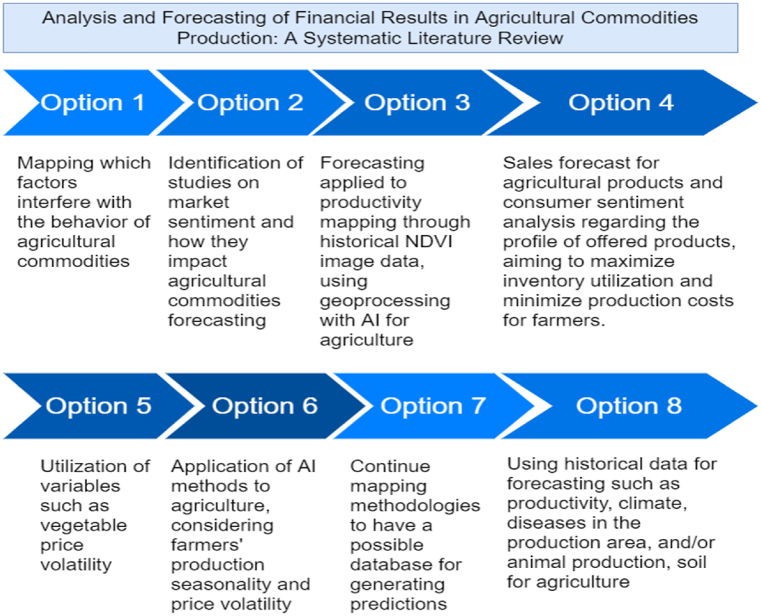
Research opportunities.

Source: Authors.

Through this exploratory literature review, we identified gaps in methods demanding further exploration. Notably, prediction methodologies emerged as pivotal for farmers' decision-making, particularly concerning uncontrollable variables such as climate and factors like exchange rates and subsidies. Given the volatility of markets, the presentation of prediction methodologies aimed at supporting decision-making becomes imperative to mitigate risks and enhance profit margins for farmers and stakeholders in the agricultural sector. Moreover, our literature review uncovered research opportunities, synthesized in Fig. 13, derived from the analysis of articles within the final portfolio. These opportunities may pave the way for future investigation.

In Fig. 13, the main opportunities for future research are identified as follows: (i) mapping the factors that influence the behavior of agricultural commodities; (ii) identifying studies on market sentiment and how they impact agricultural commodity forecasting; (iii) forecasting applied to productivity mapping through historical NDVI image data, utilizing geoprocessing with AI for agriculture; (iv) forecasting agricultural product sales and analyzing consumer sentiment regarding the profile of offered products, to maximize stock utilization and minimize production costs for farmers; (v) utilizing variables such as vegetable price volatility; (vi) Application of AI methods for agriculture, considering farmers' production seasonality and price volatility; (vii) continuing to map methodologies to establish a possible database for generating forecasts; and (viii) using historical series for forecasting such as productivity, weather, diseases in the production area, and/or animal production, soil for agriculture.

## 5 Conclusions

This article presents a literature review of the time series forecasting methodologies for agribusiness. Using the methodology proposed by Ref. [20], with automated text mining methods (LDA) described by Ref. [21], resulting in a semi-automatic approach. The general objective was to automate the identification of relevant topics and create a final portfolio that would allow the extraction of methodologies used in the articles, as well as other bibliometric analyses. The LDA methodology extracted eight topics (k = 8). The predominant topic was identified as topic 8: *Forecasting Methods Applied to Agribusiness Time Series*.

While this study presents a comprehensive review, several limitations can be noted. The first limitation concerns the number of studies identified in the literature, likely due to the specificity of the search terms and the time frame adopted for the analysis, particularly in relation to forecasting within the agribusiness sector. Despite the significance of agribusiness, relatively limited attention has been given to forecasting studies in this area. A second limitation pertains to the scope of the databases used; the search was conducted in only four databases, which may restrict the generalizability of the findings to other commodities. Additionally, the use of the LDA approach introduces sensitivity to initial choices, such as the selection and preprocessing of texts. Moreover, as LDA is a semi-automated data mining method, it may risk missing nuanced insights, particularly in areas that require more in-depth interpretative analysis.

This literature review allowed the identification of methodologies that may assist farmers in decision-making. Scenario forecasting for agricultural commodities and enhancing the decision-making process by anticipating market risks are important tools for maximizing profit margins. It is worth noting that the results obtained are specific to the context of this research, focusing on agricultural commodities. For future research, it is recommended to address topics such as hybrid methods, utilization of preprocessing methods, optimization methods, and the influence of other variables on agricultural commodities, as well as the combination of hybrid methods with other relevant variables.

## CRediT authorship contribution statement

**Luana Gonçalves Guindani:** Writing – original draft, Methodology, Investigation, Formal analysis, Data curation, Conceptualization. **Gilson Adamczuk Oliveirai:** Writing – review & editing, Methodology, Conceptualization. **Matheus Henrique Dal Molin Ribeiro:** Writing – review & editing, Formal analysis, Conceptualization. **Gabriel Villarrubia Gonzalez:** Writing – review & editing, Funding acquisition. **José Donizetti de Lima:** Writing – review & editing, Methodology, Investigation, Formal analysis, Conceptualization.

## Data and code availability statement

The data used in this study is available in https://github.com/luanagonca937/-Trends-in-Agricultural-Commodities-Forecasting-Statistical-and-AI-Methods.-

## Declaration of competing interest

(X) The authors declare that they have no known competing financial interests or personal relationships that could have appeared to influence the work reported in this paper.
